# Philadelphia Obstetrical Society

**Published:** 1890-02

**Authors:** J. M. Baldy

**Affiliations:** Secretary; 328 South Seventeenth Street


### PHILADELPHIA OBSTETRICAL SOCIETY.

December 5th, 1889.

Dr. Wm. H. Parish reported

### TWO CASES OF CÆSAREAN SECTION.—POST MORTEM MATRIS.

In the summer of 1884, Mrs. ——, æt. about 23 years, living a few doors from where I then lived, had nearly reached the full period of pregnancy. She was under the professional charge of Dr. Wharton Suckles, who was temporarily out of the city. The patient had enjoyed excellent health prior to and during her pregnancy. She had just eaten a hearty midday meal, when on, arising from the table and passing into the parlor, she was seized apparently with intense dyspnœa, and attempted to reach a window, when she fell over on the carpeted floor, unconscious. There was no convulsive manifestation. The face became puffed and livid, with escape of frothy and slightly blood-tinged mucus from the mouth. Dr. Hickman, living about one block away, was at once summoned and hastened to the house. He found the patient moribund. In a few minutes she was dead. Dr. Hickman continued efforts at resuscitation some minutes after her death, when, finding the fœtal heart beat distinctly and nearly normal in frequency, he directed his attention to the delivery of the child. Cæsarean section was peremptorily forbidden by the distracted husband, when Dr. Hickman hastened to his home for his obstetric forceps. As he was returning to his patient he requested me to accompany him. On my arrival there was not the slightest doubt as to the death of the mother. A vaginal touch showed the cervix undilated. The palpation evinced a large child. Delivery by the vagina would have doubtless occasioned too great delay. We were repeatedly forbidden by the husband to deliver by section, and some time was lost in securing his consent, or rather non-interference. In the mean time the fœtal heart-beat had become reduced to eighty per minute, but yet remained strong and distinct. In fact I never heard, on any other occasion, so loud a fœtal heart-sound, although the lady was stout, with thick abdominal walls.

We estimate that it was about twenty-five minutes after the mother's death before permission to make a section was granted. As I still held my knife in my hand I made a rapid section, and in two strokes the fœtus was removed. The incision was a median one.

The child weighed, probably, nine pounds. It was somewhat livid. Superficial and gasping respiration occurred with the frequency of one in every forty seconds, and the heart-beat was then forty per minute. To the hand there was no appreciable reduction in temperature.

Our efforts at resuscitation did not improve the condition of the pulse or of the respiration. The child lived seven hours. Its condition was that of asphyxia, gradually induced. Oxygenation of its blood had ceased with the mother's death, and the fluid had doubtless become surcharged with carbonic acid and other gases arising from the metamorphosis of its own tissue, and possibly from *post mortem* changes in the maternal structure.

I am permitted by Dr. McElroy to report the following case: On August 30, 1889, Mrs. D. had reached the seventh month of gestation, when death occurred from protracted phthisis. The mother was greatly emaciated, and her death had been anticipated by Dr. Graham, her physician. Under the directions of Dr. Graham the abdomen was enveloped in hot flannel cloths, immediately on the death of the mother, and he had been previously summoned. Unfortunately Dr. Graham was not at home, and much time was lost in securing the presence of a physician. About two and a half hours after the mother's death Drs. Tracy and McElroy reached the patient, and finding pulsation of the fœtal heart, Dr. McElroy at once made a Cæsarean section. The abdominal incision was made to the left of the median line. The child opened its eyes and breathed imperfectly, but died in about four hours. It was of small size even for a seven months' child. Prior to the section the fœtal heart-beat was very distinctly heard, being eighty to the minute. According to what would seem reliable information, the section in this case was made about two and a half hours after the death of the mother.

Cases of *post mortem matris* delivery are of sufficient infrequency and of sufficient interest to justify publication, though they may present no new facts. There is nothing very peculiar about either of these two cases. It will be well, however, to note that a seven months' fœtus was alive when removed two and a half hours after the death of its mother from protracted phthisis pulmonalis. It is generally believed that, under such circumstances, the fœtus dies either before the mother or almost immediately after her death. In my case the mother seemed in excellent health up to the moment of death; the fœtus was a large one and nearly mature; yet it was so asphyxiated and poisoned at the expiration of twenty-five minutes after its mother's death that its removal at that time did not save its life. It is well to notice, also, that in each of these cases the fœtal heart-beat was distinct, slow, and regular, its frequency being reduced before delivery to eighty per minute. This reduced frequency being that of the adult female pulse should not mislead the attendant to mistaking the fœtal beat-sounds for the pulsation of the dead mother's aorta, for thereby delay fatal to the child would arise.

In my case we endeavored to effect a resuscitation by hot and cold douching, and subsequently by the Sylvester method. I have, however, no confidence in the latter method of effecting respiration in the new-born infant. The exposure incident thereto leads to serious reduction of temperature, and the muscular fatigue produced must be exhausting to the infant. I would suggest that in such cases as here reported the inhalation and forced introduction of oxygen be secured to effect the resuscitation of the child. When the maternal death is anticipated the oxygen should be secured in advance. Under other circumstances, inasmuch as the child often lives several hours after removal, oyxgen can generally be secured in our large cities, and possibly without very great delay.

I know of no more elaborate and rational consideration of the general subject of *post mortem matris* delivery than is presented in the excellent article of Dr. E. L. Duer, published in Vol. 12 of the "American Journal of Obstetrics." I can add nothing important thereto. I will, however, submit the following proposition:

1. That in every instance of death of the pregnant woman after a period of possible viability of the fœtus has been reached, delivery should be at once effected, preferably *per vias naturales*, if it is evident that delivery in this way can be effected as promptly as by Cæsarean section. Otherwise the section must be immediately made. One must not wait for the consent of a relative. It is sufficient to have no active resistance from that direction. The surviving parent should not be permitted to doom the imprisoned fœtus to death. Where it is very probable that the fœtus will die, or that it is already dead, non-delivery is unjustifiable. Only the certain death of the fœtus can justify the attendant in withholding his hands. The fœtal heart-sounds may be absent and yet the fœtus be living. It must be remembered that, with recent improvements in the management of prematurely born children, viability must be recognized as existing in the sixth month.

Delivery prior to viability, by section or otherwise, is indicated for the purpose of baptism, if the relatives or the deceased mother's clergyman so desire, for we as physicians must respect such religious rights and convictions.

DR. WILLIAM GOODELL.--I fully agree with the propositions advanced by Dr. Parish, but I should like to make one amendment to that one regarding delivery *per vias naturales*. Whenever the husband will not allow Cæsarean section, I should suggest slitting open the os uteri in various directions, and delivering the child in that way.

DR. JOHN C. DA COSTA.—While it might be possible to carry out Dr. Goodell's suggestion when the woman had reached full term and the vagina was distended and relaxed and ready for the labor, yet, at the seventh month, when the vagina is not relaxed, it would be a difficult matter, and more than the cervix would have to be cut.

### MENORRHALGIA AND MENORRSPASM.

BY G. BETTON MASSEY, M. D.

The list of pathological views that have been advanced in accounting for what is usually called dysmenorrhœa is somewhat extended, even when the term dysmenorrhœa is restricted to the uterine type of painful menstruation, excluding ovarian and inflammatory pains and true neuralgia. Those most prevalent at the present time, I believe, are, on the one hand, the mechanical theory of obstruction from stenosis or flexion, which may be called the Marion Sims theory, and the parametritis theory of Schultze. It is not sufficiently well known that this latter observer has completely upset the first or obstructive theory of painful menstruation by demonstrating that a sound may be passed, during the crisis of a supposed example of accumulation, without encountering fluid. Such a view is also weakened by the examples of stenosis and anteflexion that occur without painful menstruation. Yet Schultze's theory of parametric inflammation as a cause seems to me unsatisfactory. That it has failed of practical acceptance by those even who advocate it is shown by their adherence to dilatation as a means of cure.

In that excellent picture of painful menstruation contributed by W. Gill Wylie to the "American System of Gynecology," another pathological system is suggested,—hyperæsthesia of the endometrium. That an hyperæsthetic condition of the cavity does exist in these cases, I think any one who has passed a sound into them will admit. The exclamations of pain when the internal os is passed are most characteristic, and, in cases where a proper gentleness has been observed, must be other than normal; but I do not think that the word hyperæsthesia is sufficiently comprehensive as a designation of this condition. Dysmenorrhœa, or difficult menstruation, is also but a partial description of the occurrence. In view of these facts I wish to present in brief to this society a new conception of the condition involved in painful menstruation as it has been suggested to me by recent clinical studies; and I also desire to propose a more useful name as a designation of the condition.

Abnormally pain at the menstrual period usually precedes the appearance of the flow, or it may follow a slight show, and be succeeded by a normal flow. As a rule, there is no flow at the moment that the pain is greatest. These facts have been the clinching arguments in the obstruction theory; but do they prove it? The absence of dilatation of the cavity above the point of apparent obstruction is significant. This, coupled with Schultze's observations, is fatal to the theory. The dependence of pain upon spasm, however, is clear, and the absence of flow, or slight flow, during the continuance of the pain only shows that the spasmodic condition of the uterus interferes with the excretory duties of the mucous membrane. Gastralgia during the continuance of nervous dyspepsia, and simple intestinal colic, are analogous conditions If I am right in this matter, the use of the word dysmenorrhœa should be discontinued, as it forever suggests a mere mechanical condition. In its stead I propose the term *menorrhalgia* as a symptomatic designation that is etymologically in accord with associated terms, and does not tie us to a theory. If, again, it is believed that a given case of menorrhalgia is due to an inhibitory spasm, it should be called a *menorrspasm*.

This menorrspasm is usually accompanied by a permanently hyperæsthetic condition of the endometrium, and is often indicated between periods of a spasmodic stricture of the internal os when an attempt at sounding is made. Exactly how much of this intermenstrual stensosis is spasmodic and how much fibrous remains to be proven. The existence of the fibrous variety is, of course undoubted; but the ease with which ether relaxes many canals sufficiently to permit a dilator to be inserted indicates that they cannot be common, for of course an anæsthetic could have no effeet upon fibrous tissue.

Pathological anteflexion is also frequently found associated with menorrhalgia; but since the equal degree of this form of deviation may be found without pain, there can be no *essential* relation of cause and effect. The same may be said of chronic endometritis and metritis. The frequency of menorrhalgia and its probable cause—menorrspasm—during a chronic metritis, without any evidence of stenosis, is an additional proof of the non-mechanical nature of this condition, as the inflammation would doubtless interfere with contraction, and aggravate spasm at the same time.

In presenting these conceptions as a novelty, I do not wish to be understood as claiming the idea of spasm as connected with painful menstruation. Such a condition has been conceded all along, and is well understood by the patients themselves when they speak of "cramps." But the contractions have been supposed to cause pain, because the flow was pent up. The spasm, as an inhibition of a normal excretion, has not been dwelt upon.

Menorrspasm, in brief, may be said to be a neuro-myotic storm of the uterine neuro-muscular apparatus, which renders the excretion of the menstrual fluid temporarily impossible. Its remote causes may be traced to all those influences in modern life which hinder the proper development of animal life in young women.

The treatment of the disease is both general and local. Many cases get well after regulation of the bodily functions and the correction of imperfect hygiene, but many resist such measures. Of these a goodly proportion will yield to percutaneous applications of the galvanic current, poles being applied to the hypogastric and lumbar regions, and a current of from 25 to 50 ma. being turned on without shock. But often we must resort to local treatment; and of the nature of the local treatment that is most appropriate I have had some very positive experience,—an experience, in fact, which led to the conception of the pathological condition advocated in this paper. Forcible dilatation certainly cures many cases, doubtless by paralyzing the irritable fibres, as in fissure in the anus, and by stimulating nutrition; but it is not a sovereign remedy in a large proportion of cases. In my experience a more certain and less formidable remedy may be found in the intra-uterine action of one pole of a galvanic current,—usually the negative pole,—when a promotion of flow is desired with a current varying from 15 to 50 ma. *pro re nata.* A few such applications, during one or two intermenstrual periods, has cured a number of cases in my hands. A typical case was that of a young French girl of 24, who had been menorrhalgic since puberty, becoming much worse during the year preceding her application for treatment. She was badly constipated, and I at first expected to relieve her by correcting this, but her next menstrual period was as bad as ever. Examination then showed a small uterus with healthy surroundings. The sound could not be passed beyond the internal os. Twenty-five milliampères, positive, were given for two minutes with the electrode in this position. Six days later the same instrument went to the fundus, without the use of a tenaculum, and forty milliampères were given. This was followed by an easier flow than for several years. Two similar applications were made during the next intermenstrual period, followed by a painless flow. Since then five menstrual periods have passed, all normal and free from pain.

Among my notes of married women treated in this way for menorrhalgia, three who were apparently sterile have become pregnant and borne children.

As contrasted with forcible dilatation this method is simple, does not require an anæsthetic and may be employed in young girls without the use of a speculum.

DR. A. GRAYDON.—I have at present under treatment a young girl who two years ago was operated upon by forcible dilatation. She is one of two typical cases, both of which were dilated. In the one case the dilatation was perfectfully successful; in the other the operation did not effect the result expected. The girl still suffers severe pain at each period, and I have persuaded her to be put upon the electrical treatment. It is too soon to give the result. I have had curative results in sharp anteflexion both with the faradic and with the galvanic current.

DR. J. M. BALDY.—I have watched with a good deal of interest the electrical treatment as practiced at the Pennsylvania and the Howard Hospitals. If we are going to get good results from electricity in any class of cases, we may probably expect to secure them in the class referred to by Dr. Massey.

The line is drawn sharply by Dr. Massey between pathological anteflexion and normal anteflexion. In ninety-nine out of a hundred cases the anteflexion is perfectly normal. Many cases with an extreme degree of anteflexion, apparently pathlogical, have not the slightest symptoms. In other cases without pathological anteflexion, or without any displacement whatever, the dysmenorrhœa is most severe. In many such cases, I have gotten the best results by dilatation. So far as I have seen, electricity will undoubtedly relieve the pain in a certain number of cases. So will dilatation; but I take it that in the majority of cases dilatation will do the work better and quicker than electricity, and perhaps without as much annoyance to the patient.

I can illustrate this by reference to a case which was selected by Dr. Massey and myself as a test case. Dr. Massey thought that there was a pathological anteflexion; I thought that it was normal. She had had dysmenorrhœa for years. She had come from a neighboring State, where she had had dilatation performed on a physician's table, without ether. We all know how unsatisfactory and incomplete that is. She had no premonitory symptoms at the menstrual period following the dilatation, and no pain during menstruation. The relief was absolute for three months. The pain then began to return, and she came to us one year afterwards. She was given electrical treatment, twenty-five to fifty milliampères being used at two sittings. She suffered premonitory symptoms for ten days. The flow came on without pain, and continued two days without pain. She then had severe pain for one day, but during the rest of the priod there was no trouble. She had two sittings, but the relief was only partial. With the dilatation she had one sitting, and this without ether, by no means as thorough as what we understand by rapid dilatation, and the relief was absolute for three months. If this woman were put under ether and full dilatation made, the relief in all probability would be absolute and permanent. The same result could not be accomplished with electricity under several months' treatment.

DR. J. PRICE.—My experience scarcely corresponds with that of some of the gentlemen. I am not in the habit of dilating, nor have I passed a sound for some time, nor am I in the habit of jumping to a conclusion as to the necessity of treatment without recognition of a lesion. There is scarcely a married woman who has borne children who has not suffered with dysmenorrhœa, due to one of the conditions under consideration. If she had consulted a physician she would probably have been treated by dilatation or electricity, as few ever eseape. This I could demonstrate by the history of wives of young physicians. In many of these cases operation had been suggested, yet a few months later they married and became mothers. Without the presence of some positive pathological condition, I do not see why we should submit these women to operation, for surely the indications are not clear, and you have no trenchant argument for local treatment. The more I keep my hands off the better am I satisfied with the result. From no small experience, I am satisfied that many of these women suffer serious troubles from too much treatment; some suffer from too much closure of cervix, too much dilatation, too much intra-uterine treatment, and too much electrical treatment, and later submit to abdominal section for no minor trouble in the pelvis, clearly due, in many cases, to vicious treatment. I can report a large number of sections done after closure of the cervix and after dilatation. I have removed large pus-tubes one week, one month, one year, after dilatation, and after closure of the cervix.

I will present these specimens to fortify my remarks. This pus-tube, larger than the uterus, was treated for two years with a pessary and other forms of treatment. It was mistaken for a retroflexion. I examine a large number of women and fail to find pathological anteflexion, except in the presence of these conditions. If you find a pathological anteflexion, you will find a pathological lateral or bilateral trouble which contra-indicates intra-uterine treatment. In the fourth volume of Pepper's "System of Medicine" a case is cited in which the cervix was closed, and the author of the article a few weeks later removed pus-tubes. A few days ago I removed a small dermoid tumor from a woman who had suffered for years. She lay in a hospital for eight months, and submitted to seventy applications of the actual cautery for sciatica, and yet no vaginal examination had been made. In this case there was a clear indication for examination.

DR. WILLIAM L. TAYLOR.—I fully agree with Dr. Price as to the number of cases of dysmenorrhœa, but will also add the cases of young girls who are confined to bed every month for from one to four days. I would ask Dr. Price what is his treatment for such cases ? These cases are numerous and require some rational treatment. We can divide cases of painful menstruation into two classes,—the one due to obstruction, the other to excessive ovarian congestion. In the first class, dilatation, and in the second, galvanism with counter-irritation, has given me good results.

DR. J. PRICE.—I frankly say that I am afraid of the treatment, and have just reasons for being afraid. I wish to fortify the position of Dr. Goodell, one that he has held for a long time, although he practices these methods freely. Dr. Goodell has frequently called our attention to these moral rapes, and I wish to say that this is done too often, particularly by our female friends. Sex surely does not sanction the examination of every young girl with backache.

DR. WILLIAM L. TAYLOR.—That does not answer my question. There are a certain number of cases that are confined to bed every month, and in which the bromides, opium, and chloral are largely contra-indicated. The only method in such cases is to make an examination, and to use such positive operative measures as will relieve the trouble.

DR. JOSEPH HOFFMAN.—I have under observation such a patient as Dr. Taylor has spoken of. I have treated her for a number of months, but have yet not made an examination. The girl is sixteen years of age, and has pain for three or four days at every period. I advise going to bed and the use of hot fomentations. This is done for two or three days, and for the rest of the month she does very well. Where the girl is anæmic and neurotic, these conditions are very likely to lie at the bottom of the trouble. In these cases the best treatment is rest.

So far as intra-uterine applications are concerned, I think that there is no doubt that they are abused. Those who are called upon to treat painful micturition in males from bladder trouble would not feel justified as soon as a man came with such disorder to pass a bougie at once. We are not to suppose that because there is pain there is stricture. Pain may exist outside of any tangible condition, and may be cured by simple rest.

In regard to Dr. Massey's nomenclature, I see nothing in the terms suggested that has not been expressed before.

DR. JOHN C. DA COSTA.—The gentlemen seem to consider dysmenorrhœa a disease rather than a symptom. We have a variety of forms of dysmenorrhœa. There may be an obstructive dysmenorrhœa, a congestive dysmenorrhœa, and a neuralgic dysmenorrhœa. Take a sharply flexed uterus. If the canal follows a perfect curve, there is no dysmenorrhœa. If, however, there is a sharp bend, such a uterus will have to go into a mimic labor to expel the blood, and we have dysmenorrhœa. Again, the uterine canal may be large enough to pass the menstrual blood, but as the result of such passive or active congestion as may follow "catching cold" or the congestion sometimes following coition, there may be swelling of the mucous membrane which will close the canal, and the woman will suffer pain. There may be another form, due to ovarian neuralgia. In the first two classes dilatation answers the purpose; in the last, electricity may answer.

I cannot understand that peculiar formation of mind that is not afraid to cut open the abdomen, tear adhesions apart, and yet will not examine the pelvis by the vagina to see what is the matter, or is afraid to dilate a cervix. With all due deference to Dr. Price, I do *not* think that most cases of dysmenorrhœa are due to pus-tubes. I have seen a number of cases in virgins, who could not have had gonorrhœa (which some allege as the cause of pus-tubes) and who did not have pus-tubes. I have not been afraid to handle these cases, but have treated and cured them, and there has been no after trouble.

DR. WILLIAM GOODELL.—As, in a certain sense, I have been the parent of dilatation in this city, I should like to give my experience. I have performed the operation three hundred and fifty-four times, and barring the first few cases, in which I did not use antiseptic precautions, there has been no trouble, and even in one or two of the earlier cases, where trouble occurred, it was very slight.

I agree with Dr. Price that young girls should not be examined without due reason; but when a girl comes who has been suffering month after month and year after year, and her life is made miserable, it is only right that something should be done to relieve her. I object strongly to the frequent vaginal examinations that are made simply for backache. Where an examination is desired by the mother, I frequently make a rectal examination; this enables me to determine the position of the womb and the condition of the pelvic organs.

I think that Dr. Price sees these things from a different point of view, He has a large dispensary practice, and sees a certain class of patients who are liable to gonorrhœal trouble. He is probably right from his point of view. I have myself never seen a positive case of dysmenorrhœa from pus-tubes. I have seen painful menstruation in these cases, but the pain was in the ovarian region. It is not a uterine pain. Overalgia is by no means frequent without pus tubes.

I should like Dr. Price to explain one of his remarks. He speaks of cases of closed os uteri in which there were pus-tubes.

I cannot give up rapid dilatation of the cervical canals to electricity, unless the latter is as effectual, and it can be used without ether and at one sitting. The only objection to the operation of rapid dilatation is the necessity to give ether. A physician not long ago made the statement that he could cure any kind of stricture of the urethra by electrolysis. He was offered cases by some of his brother physicians of that city; but, if my memory serves me no trick, when he was closely challenged he failed to respond. I have seen the statement made in some of the medical journals, that the use of electricity is followed by dilatation of the uterine canal. But I do not understand the *rationale* of this agent, and I should like to hear Dr. Massey explain it.

DR. J. PRICE.—I did not attribute dysmenorrhœa to the presence of pus-tubes. What I said about pus-tubes had nothing to do with dysmenorrhœa or its treatment by dilatation or electricity, except as sequelæ. I wish clearly to condemn the indiscriminate examination of virgins. General treatment, out-door life and rest do cure these cases. It is in just these cases that Dr. Weir Mitchell recommends his rest treatment, and in which he has such success. I would refer Dr. Taylor to the collection of photographs which were sent here by Dr. Playfair. I presume that all these women suffered dysmenorrhœa, and they were all cured by rest, and, I venture to say, had no local treatment.

DR. J. M. BALDY.—Does Dr. Price mean to say that all cases of dysmenorrhœa in virgins can and should be cured by the rest treatment? I have heard him unqualifiedly condemn it.

DR. WILLIAM GOODELL.—By what authority does Dr. Price state that the cases of Dr. Playfair suffered from dysmenorrhœa?

DR. J. PRICE.—I only presume that they did. They got well with general treatment, married and bore children without electricity. Such cases, in my opinion, always have dysmenorrhœa. But few women are free from it.

DR. J. M. BALDY.—There is still the class of cases referred to by Dr. Taylor, of young girls who are not married and have no idea of getting married, and who suffer the torments of the damned at the menstrual periods. They have to go to bed for three or four days each month, and some are made worse for going to bed. These cases surely demand some treatment. It is all very well to indiscriminately condemn electricity, dilatation, and other forms of local treatment, but what does Dr. Price offer us in their stead? Practically nothing. Dilatation will cure a certain number of these cases. Every one admits that indiscriminate examinations are wrong; but the members of this society are surely beyond such abuse. If a woman has been suffering for years, is still single, and getting worse, her virginity should be no bar to treatment.

DR. WILLIAM GOODELL.—I have had a good many patients under the rest treatment, and while Dr. Price is right in regard to the majority of cases of dysmenorrhœa cured by this treatment, yet he is not wholly right. I have been obliged to dilate some of my own cases, and I have repeatedly been asked by Dr. Mitchell to dilate patients for him who had been under the rest treatment, and who have been cured in every respect, with the exception of the dysmenorrhœa.

Dr. M. PRICE—There is one point I want to discuss, and that is the examination of virgins. A short time ago I sent a patient to a specialist for treatment of the throat. He told her he could cure her in six weeks; but at the end of that time she was no better. He then told her that she had uterine trouble, he could see it in her throat, and sent her to two or three specialists, who would not examine her, there being no indication for so doing. He said he would have her examined in spite of the father and in spite of the opinions of the specialists. If it is ever necessary to examine a virgin, it should be done under ether, in the presence of more than one individual.

DR. G. BETTON MASSEY.—Dr. Baldy has scarcely treated me with fairness in mentioning a case in which the treatment is only commenced; still his relation shows considerable benefit from two applications. The paper did not claim that a single treatment would cure these cases. I distinctly stated "two or three treatments during at least two intermenstrual periods."

I am glad to see that, to-night, some of the surgeons are so conservative. While I agree with what has been said as to the inadvisability of vaginal examination in virgins, yet all must admit that at certain times examinations become necessary. I have myself, always tried other measures first, and continued them for sometime—often too long. A special advantage of this method of local treatment is that you do not have to use the speculum, and it is not necessary to dilate the vagina.

Dr. Da Costa has said that the menstrual pain is sometimes due to trouble in the ovary; I particularly excluded that from the paper. I believe it is pretty well understood that you can discriminate between the uterine pain of menorrhalgia and the ovarian pain preceding the sickness.

Dr. Goodell has made a good point in regard to the inefficiency of a single sitting of electricity. Where the trouble has to be relieved at a single sitting, dilatation comes in. I cannot explain how electricity overcomes the contraction of these canals. I have been justly charged with a mixing of facts in my book by one of the reviewers, who pointed out that in one place it is stated that there might be atresia of the cervix from the use of too powerful a current, and, in another place, the same thing is recommended to produce enlargement of the cavity. I have, myself, never seen atresia follow the use of heavy currents. Apostoli mentions its possibility, but he uses a sound that is bare at the external os, whereas, under the impression that a cauterization at the mouth is inadvisable, I try to apply the cauterization to a certain part only of the cavity. However it may be explained, I do find that under the application of either the positive or negative current to the cavity of the uterus, after one or two applications the sound passes with greater ease, and that the succeeding menstrual flow is almost invariably less painful. There are two possible explanations: the one, that the canal is rendered patulous; the other, that spasm is relieved. I do not believe that pelvic trouble can be brought on by this treatment of menorrspasm in a woman otherwise healthy, and should not hesitate in such cases to apply powerful currents to the interior of the uterus, if proper aseptic measures were used. The troubles which may follow are due rather to dirt than to the operation.

DR. JOHN S. MILLER.—In presenting the following case of supra-vaginal hysterectomy, I do not profess to tell much that is new or strange, but only desire to place it on record for statistical purpose. Statistics are valueless if failures are not reported as well as successes.

Mrs. E. B., æt. 56 years, American. Menstruation began at the age of 14, and had one child at 26. No miscarriages. Her health was always fair; from the beginning her catamenial periods were marked by an excessive flow. At the age of 23 she had a severe attack of diarrhœa, which apparently awakened a continuous metrorrhagia of such gravity, that for six months she was confined to bed. At that time, it is said, her condition was more serious than when the hysterectomy was indicated a few weeks ago. Three and one-half years ago the bleeding stopped for about four months, and the patient was happy that her menopause had at last put a check on this heavy drain and pain.

After some exposure and overwork the uterine hemorrhages again appeared with increased vigor; the pain was also greater, and in a short time she became greatly reduced. During all these years she had been treated homœopathically, and she was told that if the blood were stopped she would perish.

For the past three years the bleeding was constant, varying in degree from day to day. I saw her for the first time about three weeks ago, and found her comparatively fairly nourished, considering the history of her case. She was very anæmic, and her suffering excessive. The uterus was found to be about the size of a fœtal head, and fibroma of the uterus was diagnosed. Ergot and tampons were used with some effect to control hemorrhage during the week in which she was prepared for the operation.

As a proper preliminary precaution the condition of the heart, lungs and kidneys were interrogated and found apparently normal. After the usual preparation for an abdominal section, ether was used as the anæsthetic. A three-inch incision was made, which was afterwards enlarged sufficiently to deliver the tumor. The growth was about the size of a fœtal head, globular in form, with a number of cysts, the largest of which was of about the size of a hen's egg; this cyst ruptured during the efforts at delivery of the tumor. Tait's corkscrew was introduced into the most solid portion, but it did not take hold in the friable tissue. There were no adhesions to the surrounding structures, and with some slight difficulty the tumor was delivered through the abdominal incision. The neck of the uterus was transfixed, and a Koeberle's *serre noed* charged with a so-called Delta metal wire was applied, which twice broke. A German-silver wire was next substituted, which also gave way. Fortunately, I had provided myself with some thick rubber cord which I wound (on a stretch) tightly three or four times below the transfixion needles, and the tumor cut away. The stump was about two and one-half inches in diameter, but was shaved down to about an inch. The peritonæum was stitched over the stump. The parietal peritonæum was carefully stitched to the neck of the pedicle, and thus it was excluded from the abdominal cavity. A glass drainage-tube was used, and the blood and serum were frequently removed with a uterine syringe. Only three and one-half ounces of ether were used, and there were no complications to deal with to interfere with the prompt dispatch of the work, except the aggravating breaking of the wires. Although the patient was very much depressed during the operation, she rallied well soon thereafter. For twenty-four hours she suffered severely from retching, which was uncontrollable during this time. The patient now seemed quite hopeful, and her condition was no worse than what I had frequently observed before in simple oophero-salpingotomies. Her temperature never went above 99°. On the morning of the fifth day a decided change for the worse came over her, and no assignable cause was discoverable. She refused to take nourishment and stimulants; these were introduced through other channels, without, however, any appreciable effect. There was no evidence of hemorrhage, no strangulation. Tympany was slight. The excreta had been normal since the operation, and I was totally at a loss to understand the cause of this sudden collapse. Despite careful and active stimulation the heart became very feeble, and unconsciousness supervened, followed later in the day by *exit letalis*.

Autopsy twenty-four hours later disclosed the wound and dressings in a perfectly aseptic condition. The wound was quite firmly united, but could be separated by tearing with the fingers. The stump was dry and mumified in appearance. The peritonæum was normal, and only reddened at the line of union with the abdominal wound. The pelvis contained about two ounces of an odorless, serum-like fluid, such as we frequently find in *post mortem* examinations.

A thorough examination was not permitted, and the parts could be only examined through the re-opened wound.

To Dr. Morris Booth Miller, resident physician of St. Joseph's Hospital, I am indebted for the following notes on the microscopical examination of the tumor. The growth is a Leiomyoma. "Its principal characteristics are as follows: in size it is about as large as two fists, smooth, slightly lobulated, firm, shining on section, and is pale gray, with concentric lines when cut. Microscopically it presents the characteristic appearance of one of these tumors in its smooth, long, nonstriated muscular fibres, with a varying amount of intercellular connective tissue. Towards the interior and close to the cornua of the uterus the texture of the tumor is less firm; in fact, almost becomes cavernous in its softening, with the softened spaces filled with a mucoid degeneration. No cells of a sarcomatous nature could be found, though the fact that this growth affected principally the fundus, in contradistinction to the cervix, which is usually attacked by sarcoma, would almost of itself exclude the cancer idea.

"A word as to Leiomyoma: the usual seat of these growths is the uterus, though the testicle, prostate, etc., may also sometimes be affected. In fact, the enlarged prostate of old men is always Leiomyoma of very slow growth. The fibres are long and smooth and have a long nucleus. On cross-section the fibres are nearly circular. They may be closely bound together or may be separated, and may be of such a disordered appearance as to suggest malignancy. The size of these tumors varies from that of a fist to that of a pregnant uterus. They are usually encapsulated and, as a rule, grow slowly; though often after they have gained some size, they suddenly take up a rapid growth.

"They usually occur late in life, and seem to occur with remarkable frequency in negresses. They are perfectly benign, but they endanger life through their excessive hemorrhagic tendency.

"As to the diagnosis of these tumors anatomically, it is well-nigh impossible to identify them by any other means than the microscope, under which they present their characteristic appearance; and by the absence of any tumor resembling them, the diagnosis is made easy."

Dr. E. E. MONTGOMERY.—Sarah Perry, age 34, colored woman, native of North Carolina.

She was admitted to the Medico-Chirurgical Hospital the 14th of July, 1889, suffering from abdominal enlargement, which had been in existence for about eight years, and given her so much distress and discomfort that she was willing to undergo any risk to get rid of it. She had been married a number of years, but never pregnant. She had not suffered from hemorrhage, but from the growth and pressure of the tumor. On admission, the abdomen was very considerably distended, presenting a tumor which could be felt extending beneath the ribs in the right hypochondriac region. Otherwise her general appearance and health were good. The tumor was irregular, nodular, movable, and apparently a part of the uterus. By vaginal examination a portion of the tumor could be felt filling up the pelvis, and could not be readily dislodged. The mass was not painful, nor was any fluctuation present. Diagnosis: multiple fibroma.

The patient was so desirous of being relieved of its weight that the operation was decided upon. This was performed July 16, in which I was assisted by Drs. West, W. S. Stewart, Reyburn, Hammond and Mr. Maier. Making an incision five inches in length nearly to the umbilicus, the upper nodular mass of the tumor was secured and drawn out by inserting a corkscrew into its substance. The raising up of the remaining portion of the tumor was attended with little difficulty; the broad ligaments, however, were so short that the tumor was still held against the rim of the pelvis, so that as the next step in the operation, the finger was driven through the peritonæum close to the surface of the cervix, the ligaments grasped with large forceps, and separated from the uterus. This permitted the uterus to be raised up so that the cervix would form a stump. When it was compressed by a rubber ligature, and the tumor of the uterus cut away, the stump was then raised up, transfixed by pins, and secured by the sersenœud and fastened in the lower angle of the wound; the vessels of the broad ligaments having been previously ligated, the abdominal cavity was thoroughly irrigated, and, as there still remained some oozing, a drainage-tube was placed just above the stump. The abdominal wound was then closed by sutures, and a dressing of iodoform gauze applied. The stump itself was thoroughly dusted with iodoform and surrounded and covered by gauze. The patient suffered no shock, and the subsequent progress was exceedingly favorable. The highest temperature reached was on the ninth day, when it attained 102.4°. The patient complained of but little pain, and the discharge from the drainage-tube ended on the second day, so that it was removed. The stump was cut away at the end of the week, removing the pins and wire; following its removal there was considerable retraction of the parts, leaving a cavity to fill up by granulation. This was completely healed by the middle of the fifth week. The patient was discharged well August 19, 1889.

DR. MONTGOMERY reported a case of vaginal hysterectomy and presented the specimen with the following history:

Mrs. L., æt. 48 years, admitted to the Medico-Chirurgical Hospital, October 31, 1889. Mother of seven children; has had three miscarriages; youngest child æt. 5 years; last miscarriage occurred two years ago. Previous to its occurrence her health had been very good. Since she has suffered from frequent hemorrhages. Three times during this period she has missed one menstrual period. For a month before her entrance she had a continuous bleeding, so severe at one time as to require tamponing of the vagina.

She was seen a couple of weeks before admission, in consultation with Dr. M. J. Cummings. The uterus was enlarged and retroverted. The cervix was large and lacerated, the posterior lip abraded and bleeding. The os presented a number of small granular nodules. The uterus was drawn down and a small section of the mucous surface removed, which was examined microscopically by Professor Laplace, who without hesitation pronounced it epithelioma.

As the uterus was readily movable, and careful rectal examination failed to disclose any involvement of the broad ligaments or surrounding tissues, extirpation of the organ was advised. This operation was done November 2.

After careful irrigation with acid sublimate solution, the patient was placed in the recumbent position, the uterus exposed with lateral vaginal retractors, seized with valsellum, and with a knife separated by a circular incision from the vagina. The anterior and posterior surfaces were pushed off until the peritonæum was reached. The latter was opened behind and a large sponge introduced. The anterior was opened, and clamps applied on either side the uterus to the broad ligaments. The uterus was now cut away between the clamps, and after the removal of the sponge a wad of iodoform gauze was inserted.

Duration of operation, twenty-five minutes.

The immediate effects of the operation were profound shock. Subsequent temperature was high, reaching 103° about the eighth day.

The clamps were removed at the end of twenty-eight hours.

The patient left the hospital at the end of three weeks, feeling strong and well.

The uterus was opened and it was found that the whole mucous membrane was in a state of malignant degeneration.

Although the appearances prior to the operation, indicated disease confined to the cervix, the subsequent examination demonstrated the futility of any operation other than extirpation.

Dr. B. F. BAER reported two cases of vaginal hysterectomy for epithelioma of the uterus.

I have had two cases of vaginal hysterectomy for cancer during the year, which I wish to place on record in connection with the case which has just been reported.

Mrs. H. was brought to my office by Dr. J. P. Pyle, of Wilmington, on February 1, 1886. She was 36 years of age; married; had six children. The last child was born seven weeks before I saw the patient. The labor—which was premature, seven and a half months—was difficult but not instrumental. The patient stated that she suffered unusual pain during the first stage of labor, cutting and tearing in character; then the child was expelled suddenly, considerable hemorrhage attending the expulsion. The child died ten hours afterwards. She remained in bed not longer than the usual period after the labor, and then went about feeling as well and strong as after her former labors. So far as she knew she had been perfectly well until some time in October, when she began to have a profuse watery and fetid discharge from the vagina. She did not have any pain or hemorrhage until the onset of labor, nor did she have any unusual flow after the labor, the main symptom of which she complained being the fetid watery discharge.

For this she consulted Dr. Pyle about a week before I saw her, who correctly diagnosticated epithelioma of the cervix.

On examination I found a cauliflower-like mass as large as my fist distending the upper portion of the vagina and growing from the neck of the womb. The nodules were not friable and no hemorrhage attended the examination. The body of the womb was greatly enlarged, but it was apparently perfectly mobile. The tissues exterior to the uterus were free from infiltration so far as could be determined. The vagina was apparently not involved.

In view of the above physical condition I advised hysterectomy, and the operation was done two days later, on February 3.

I began the operation by removing the degenerated tissue with the curette and scissors. This was necessary to gain room and to protect against infection, the parts being constantly irrigated with a 1 to 4,000 solution of the bichloride of mercury. I then proceeded by cutting through anterior to the uterus. The vessels were large and the tissues soft, spongy, and vascular, as was to be expected seven weeks after labor, when involution had not been entirely completed. After the peritoneal cavity was opened I passed in two fingers and enlarged the opening by tearing from side to side. I then cut through posteriorly. I did not cut at the side of the cervix because it was now found that the vaginal wall was involved to a slight extent at this region on either side, and for the further reason that the parts were so vascular that free bleeding occurred when a cut was made. One blade of Doléris' clamp was then passed along the posterior surface of the right broad ligament, and the other anterior, and made to grasp the uterus as close to the side of the pelvis as possible. The lower part of the ligament was then severed with scissors so far as it was grasped by the clamp. This gave more room, so that I was enabled to pass my finger within and over the Fallopian tube, when I found that the latter was not grasped within the clamp. This was now secured with a Wells' pedicle forceps and then severed. I now passed the second clamp over the left broad ligament, and began cutting on the other side in the same way as far as I could reach with the scissors. By great effort the uterus was then pulled down outside of the vagina, when it was finally severed. There was some general oozing, but I did not lose any time in trying to ligate vessels, as there did not seem to be anything but this general oozing. I packed very firmly with iodoform gauze.

The patient stood the operation well, but died four days afterwards from peritonitis.

My second case was not an operation of election.

I saw Mrs. S., a patient of Dr. L. P. Reiman, in February, 1889. She was 49 years of age; married; had two children, the last one nineteen years ago, after normal labors. The menopause had not yet occurred.

She had enjoyed remarkably good health all through her life until the following symptoms began to develop:

One year previous to the above date she found that she was flowing more freely at her periods, and during the previous six months she had had several attacks of severe flooding. During this time she began to lose flesh, and had lost probably thirty pounds; but she still presented an appearance of ruddy health, without the slightest sign of cachexia.

Examination revealed the cervix to be the seat of a carcinomatous growth, cauliflower in form, and involving slightly the left side of the vagina. There was no evidence of infiltration of the lymphatics or the other tissues outside the womb, that organ being mobile, except at the point of involvement of the vagina, where the infiltration appeared to extend deeper than the tissues of that organ. For this reason especially I advised simply curetting and cauterization, for the purpose of modifying the hemorrhage and prolonging the patient's life. During the operation I accidentally punctured the posterior wall of the vagina, and my finger entered Douglas' cul-de-sac. I then decided to remove the uterus, and I therefore hooked my finger over the body of the organ and brought it down through the opening which I had inadvertently made. The broad ligaments were ligated with silk ligatures and the vagina tamponned with iodoform gauze.

The patient made a good recovery from the operation, and for a time was greatly benefited; but a letter received to-day from

Dr. Reiman informs me that the disease is returning at the side of the vagina, and that the patient is greatly emaciated and in a very bad condition generally, having a cough and hemoptysis also. She will probably die within a short time.

Basing my opinion upon my own experience I am inclined to look with disfavor upon this operation. I, of course, at the same time recognize that these are not proper test cases. I believe if I were to be called upon to-morrow to decide in a case of epithelioma of the cervix, the body of the womb not being involved, I should advise the high amputation of the neck, and not hysterectomy. I have a number of cases in my mind now where amputation of the cervix, simply, has resulted in apparent cure of the disease, for after several years—in one case four or five years—the disease has not returned. Of course if it were found during the operation that the disease had extended to the body of the womb it is likely I should complete the operation by removing that organ, provided there was not infiltration of the tissues outside.

DR. GEORGE E. SHOEMAKER.—It would be interesting in all cases to know the results several years later, but especially after vaginal hysterectomy. Hofmeier has published statistics showing that the duration of life after vaginal hysterectomy is less than after the various partial operations, such as Schroeder's. After the lapse of four years not one case on which vaginal hysterectomy had been performed was living, while forty-one per cent. of the cases which had had some operation similar to that described by Dr. Baker were still in good health. All the cases were operated on by the same man, at the University Frauen Klinik, in Berlin. The total number of cases was one hundred and twenty-nine.

It seems to me that it cannot be certainly shown that the lymphatic glands high up are not involved. If all the diseased tissue is not removed vaginal hysterectomy is no better than the so-called partial operations. If you get the case in the early stage, the partial operation is thorough; but if the case is not seen in the early stage the most thorough operation is not sufficient.

DR. G. BETTON MASSEY.—It may be of interest in this connection to refer to the electrical treatment of cancer, in regard to which there has been considerable hopeful expression in England. I have tried this treatment in two cases of cancer of the cervix. One was seen a year ago in January, with a cavity two inches in diameter, involving the whole cervix, the uterus being fixed and the pelvis infiltrated. I persuaded her to go to a surgeon, but he declined to interfere. She was then put upon positive galvanic-caustic applications to the cavity. The current's strength was usually 150 milliampères. The result was that the hemorrhage immediately ceased. The horrible odor was, in the course of four or five applications, almost completely removed. Appropriate washes were used at the same time, but these had had no effect before. They brought away large quantities of white crumbly material. The treatment was continued three times a week for three months, the extreme pain of which she complained being relieved for twenty-four hours after each application. At the end of that time the cachexia was not improved and a cavity with hard edges existed where the carcinoma had been present. She was then advised to go to the country, where she died three months later. This case had lasted a year and a half when I saw her first. The best that can be said of the treatment is, that it palliated the symptoms most remarkably, and possibly prolonged life.

In the second case the disease is not so far advanced. One treatment has stopped the hemorrhage and relieved the pain to a great extent. The case is still under observation.

DR. JOHN C. DA COSTA.—I would suggest, that where the epithelioma is confined strictly to the neck of the uterus the high amputation will answer the purpose in cases where there is sound tissue that can be reached above the diseased mass. By cancer, I do not mean supposed cases, or cases of mere erosion or ulceration, but those in which the history, symptoms, appearance, and the microscope all pronounce it cancer. Some five years ago I began the thorough and high amputation, and have done the operation several times, and in no case so far has there been a return of the disease. I make a wedge-shaped incision in the sound tissue, carrying it up into the body of the uterus until healthy tissue is reached. This is known by the appearance of the tissue and the diminution in the bleeding. There are then two modes of proceeding: one that I have used was suggested by the president of this society, and consists in the application of a mixture of bromine, iodine, carbolic acid, and alcohol; the second method is to stretch the mucous membrane across the wound as far as possible and tack it together. When the caustic is used, it is necessary to protect the vagina, and the case has to be treated every two or three days. Where the mucous membrane is drawn over the wound there is no more trouble than after a simple amputation of the cervix. There has been recovery in every case. I offer this as a procedure which might be employed in cases where hysterectomy might imperil the woman's life. Where the pelvic glands are involved no operation will cure.

DR. LONGAKER.—Some years ago the late Marion Sims advised the use of a strong solution of chloride of zinc. In the *American Journal of Obstetrics*, in 1884, Dr. Van de Warker enlarged upon this method, and reported a number of cases. Four or five years ago, I operated by this method in a case of large epitheliomatous mass of the cervix. This was removed with scissors and curette and the cavity filled with cotton saturated with a fifty per cent. solution of chloride of zinc. After some days a slough extending up into the cavity of the uterus came away. The result was that the woman was completely cured. I saw her a year ago, and there was no return of the disease.

DR. C. P. NOBLE.—I wish to speak of some points in the technique of vaginal hysterectomy. I removed the uterus for epithelioma of the cervix by vaginal hysterectomy last Saturday for the first time. I clamped one side and cut the uterus away. There were some vessels which began to bleed after the clamp was applied. I tied off the opposite side in the usual manner. Examining the clamp on the left side I found that it was not holding nicely, and feared to trust to it. There was considerable oozing. After consultation with Dr. Kelly, I concluded that it would be better to remove the clamp and ligate the vessels on that side, the large ones separately and the remainder by a quilted suture running fore and aft through the broad ligament. This was difficult, and required considerable time. The patient has, however, done nicely.

It seemed to me that this operation could be simplified. Like many other operations, vaginal hysterectomy is, in the beginning, more or less exploratory. After separating the vagina with the finger in connection with the connective tissue surrounding the uterus, one can form a better idea whether or not the glands are involved. It seems to me that instead of following the usual procedure, it would be better to ligate off to the neighborhood of the internal os, first on one side and then on the other, and explore the base of the broad ligaments on each side. If we want to use the clamp, we shall not have to include so much tissue, and the operation would be simplified. Should the broad ligaments be found infiltrated, the operation, done after this technique, could easily be completed as a high amputation of the cervix.

DR. BARTON C. HIRST.—I saw Winckle, in Munich, do an operation of this sort in a way which I think would, in some cases, simplify the procedure and prevent serious hemorrhage. He passed a curved needle around the uterine artery on each side, and then separated the uterus from its vaginal attachments. He next split the uterus in two, and removed each half separately.

DR. M. PRICE.—In Dr. Miller's case, the woman had prepared herself for death, and made all the arrangements for her funeral. That is a strong argument against operation.

DR. J. PRICE.—I witnessed the operation in Dr. Miller's case. As far as the operation was concerned, every detail was perfect. Possibly drainage would have prolonged her life. There were some bowel adhesions on the left side. The presence of a few drachms of serous liquid is, I think, sufficient to explain the death on the fourth day.

DR. JOHN S. MILLER.—We did have drainage in this case, and the wound was in a perfectly aseptic condition. I am satisfied that the serum found was due to *post mortem* changes. The pelvic cavity was thoroughly drained. The adhesions referred to were very slight and readily broken with a sponge. The patient was four years older than she claimed to be. This may have had something to do with the result.

DR. E. E. MONTGOMERY. . . . —In reference to the results of these operations, I would state that I have performed four operations of this character. Three of these patients are living. The first operation was done in October a year ago. I had the opportunity of examining this case in the latter part of August of the present year, and there were no signs of a return of the disease. The fatal case did well until the eighth day, when tetanus set in, proving fatal on the fourteenth day.

In one of the French journals of the present year will be found reported four hundred and ninety-five cases of vaginal hysterectomy by Maurice Hache, and he compares the results with those obtained by other operations. His results have been better than those obtained by the high amputation of the cervix, as recommended by Baker. He counts no case a cure that has not lived over two years. Twenty-six per cent. of his cases have lived this time. After amputation of the cervix, only twenty-two per cent. had lived this long. Under the old methods the danger of the operation was much greater than at present. The duration of the old operation was from one and a half to three or four hours. Under present methods the operation can, in favorable cases, be completed in fifteen to thirty minutes.

Munchmeyer has reported a number of cases, an account of which appeared in the "*Medical News*" of last September. He operated in eighty cases, with only four deaths. In the cases reported by Maurice Hache, one case was living at the end of fifteen years.

Considering the difficulty of determining whether or not the interior of the uterus is involved, and the experience of general surgeons that in cancer of the breast the axillary glands should be removed, it seems to me that where the uterus is the seat of malignant disease, its complete removal is the preferable operation, and offers the greatest certainty of eradicating the disease.

It is true that we cannot follow the disease into the pelvic glands, as we do into the axillary, but we can determine whether or not the disease has passed into the broad ligaments. Where the disease involves the broad ligament and the vagina, I should say that extirpation was not justifiable.

I am glad that Dr. Massey is experimenting with electricity in these cases. Cancer of the uterus is a disease of such grave character that we should certainly encourage every investigator to discover methods for its eradication, its relief or palliation.

I cannot see why there should be any hemorrhage with the use of the clamp. If properly applied, the clamp closes all the vessels, and there can be no bleeding.

DR. NOBLE.—Operation for the restoration of the sphincter-ani in certain cases of laceration of the perinæum.

A not uncommon result of operations for the restoration of the perinæum, when that structure is torn completely through into the rectum, is that the operation is a failure, in so far that the torn ends of the sphincter-ani fail to unite. The most important end sought is not obtained, and must be brought about by a subsequent operation.

The method heretofore employed, so far as I am aware, has been to denude more or less of the rectal and cutaneous surfaces of the perinæum, special attention being paid to freshening the torn borders of the rectum and ends of the sphincter-ani muscle, and then to unite these tissues as in the original operation.

On the 13th of October I saw, in consultation with my friend, Dr. J. N. Boyd, Mrs. F., æt. 23, who had given birth to a child about nine months before. The labor was difficult, and was terminated by the use of forceps. The woman sustained a laceration of the perinæum, extending into the bowel. This was sutured the following day by her attendant. Union resulted, with the exception of the torn ends of the sphincter-ani muscle. It occurred to me that the principles of the flap-splitting operation could be easily applied to this condition, and on my recommendation it was done by Dr. Boyd. A transverse incision was made close to the rectal border of the perinæum, extending half an inch up the bowel. A lateral incision was made in a backward and outward direction, from each end of the first incision, to lay bare the severed and retracted ends of the sphincter-ani. The lateral incision was extended up the bowel as far as the upper border of the sphincter muscle. The loose flap thus formed within the lateral incisions and beneath the transverse incision was to be turned into the lumen of the bowel. (The flap was accidently torn in the middle line, necessitating three rectal sutures.) The flap being turned into the bowel with tenacula, and the ends of the sphincter brought forward with bullet-forceps, sutures were introduced after the usual manner. Special care was taken to introduce the sutures sufficiently far back to catch the sphincter muscle, after the manner of Emmet. Silkworm gut and silk were used. The result was a perfect cure.

The method was so simple and satisfactory to Dr. Boyd and myself, and the result obtained so perfect, that I have concluded to report it to the society.

Dr. WM. GOODELL gave the following history of a case of burst purulent appendages:

M. D., aged 39, single, had an attack of scarlet fever in childhood, since which she has never been well. Having pelvic and ovarian pains early last year, she was treated by an excellent physician with electricity to ovaries and glycerine tampons to vagina. On June 17th, 1888, two days after such a treatment, she had a very serious attack of "general peritonitis," which her physician referred to a burst cyst. She was six weeks in bed and had a narrow escape from death. After this her sufferings were much greater, and she was sent to me. Finding that she was too sore to be satisfactorily examined without ether, I anæsthetized her on October 9th, 1889. The womb was found absolutely fixed, and an irregular tumor to one side of it suddenly collapsed under the bimanual pressure. For two days she had high temperature, rapid pulse and abdominal pain. October 9th, I operated on her before the ward class. On opening the thickened peritonæum a large amount of grumous and dirty fluid escaped. It came from the collapsed right ovary. Both tubes and the remaining ovary were filled with the same grumous fluid. They were desperately adherent,—had fairly to be dug out; during their removal each one burst, adding its quota to the amount of foreign fluid in abdominal cavity. The womb being adherent to the right side of pelvis, was released by slow peeling off its coat of false membrane. Four large jugs of water were needed to cleanse the peritoneal cavity, and a drainage-tube was put in. The patient recovered promptly.

DR. J. PRICE, *report of cases*:—

I have to present, first, an interesting fresh specimen, a large pus-tube and abscess of the ovary, right side. The husband now under treatment for gonorrhœa. The abscess of the ovary has completely disorganized the gland. This is a very common result in abscess of the ovary. In some old pelvic abscesses it is impossible to find even a semblance of the ovary or ovaries. In this case there was a general peritonitis, extensive bowel adhesions, the left side remaining apparently healthy. It is a typical case of unilateral orchitis or salpingitis and ovaritis, resulting in abscess of both tube and ovary. It demonstrates beautifully how common it is to find multiple abscesses in the pelvis. Again it shows the folly of the vaginal or Martin treatment by incision and vaginal drainage. It only adds great complications to the surgery that must follow to cure these cases. This I have practically demonstrated recently and many times in my own work. In short, I dread a section in a case that has had vaginal drainage. It is the most difficult and trying of all pelvic operations. This operation was done by my brother at four this afternoon. To save lives in these cases we must work like the Fire Department. This is the third case done for gonorrhœa in the past few weeks. All the husbands under treatment at the time of the section, two of them for epididymitis.

This large tumor is an electrical tumor. It was so fully liquified that six quarts of fluid drained from the tumor into the tube I placed it in for the purpose of acertaining the amount of leakage. It is a large cystic fibro-myoma of the uterus. The cervical portion is mostly fibroid, and the fundus myomatous. The tumor has been electrified three times a week for eleven months by a pupil of Apostoli, a Boston man. The improvement was marked by, first, hemorrhage lessened, but a very maked increase of a watery discharge; second, rapid enlargement of the tumor, causing dyspnœa and excessive pressure symptoms; and, third, by a rapid emaciation and decline in general health of the patient. She demanded its removal. Operation was declined by numerous operators in Boston and New York, one operator giving her one chance in four for survival. Sometime ago I stated that surgeons would demonstrate the "electrical cure" with the specimens of the patients so treated in their hands. This in cases of extra-uterine pregnancy, fibroids, and tubal and ovarian disease. These cases are now rapidly coming into surgeons' hands, and demonstrate that such cases are among the most difficult in abdominal surgery to deal with, because of complications due to delay and mischief incident to the application of electricity. It is most lamentable and trying that we should have to deal with such grossly neglected and barbarously treated patients. Another point, I have frequently said that advanced fibroid disease is always complicated by tubal and ovarian disease. Rarely recognized before operation, this is proved by the published cases of Keith and others. Even pus-tubes are common, hence additional risk in the application of electricity when such complications generally exist. In this case I found an adherent ovarian cyst on the left side, necessitating the enucleation of the sigmoid for about four inches. There was a small cyst in the broad ligament on the right side.

It is also the first case I ever had where the broad ligament was on the posterior side of the tumor.

I dealt with the tumor by extra-peritoneal supra-vaginal hysterectomy. The pedicle was, at the beginning of the operation, about four inches in diameter, and diminished in size during the operation, until it reached about one inch in diameter. The capsule on the anterior side was transversely incised high up, and retracted about four inches, permitting the fundus of the bladder and the broad ligament to retract, so that the noeud might be applied at a higher level with less drag on the pins. The stump was fixed in the lower angle of the incision, the parietal peritonæum was stitched to that of the stump below the wire, the abdomen closed, and a dry gauze dressing applied. The patient has had no shock, no hemorrhage, and now, on the eighth day, is a comfortable, happy woman. No operative disturbance of any kind.

J. M. BALDY,
328 South Seventeenth Street. *Secretary.*

